# Hepatitis C Genotypes in Hemophilia Patients in Iran

**Published:** 2011-02-01

**Authors:** M Hamidi-Fard

**Affiliations:** 1 Student of Virology, Ahvaz University of Medical Sciences, Ahvaz, Iran

**Keywords:** Hepatitis C, Genotype, Hemophilia, Iran

Dear Editor,

I read the original article by Keshvari et al. published in the previous issue of the Iranian Red Crescent Medical Journal with interest.[1] At first, the large number of samples (367 samples) was impressive. Although, sampling and genotyping performance of such large-scale specimen need many hours, the obtained results could reflect a clear view of distribution of HCV genotypes among Iranian patients with congenital bleeding disorders.

Keshvari et al. used nine specific primers for HCV genotyping. This method can detect the mixed genotypes, however is very laborious because of its complications, i.e. each sample needs preparation of nine PCR reaction tubes. The sequencing method is the gold standard for HCV genotyping and consequently the author should have randomly compared their results with sequencing.[2]

They concluded that genotype 1a, followed by 3a were the most detected HCV genotypes in Iranian patients with congenital bleeding disorders. Their findings are concordant with the results of similar studies on HCV genotypes in Iranian HCV infected patients.[3][4][5][6][7]

According to this fact that the HCV genotyping has an important role in clinical management of hepatic patients and selecting an antiviral therapy, determination of HCV genotypes and optimization of its procedure as a routine test in a regional laboratory may be helpful. So, such research has epidemiologically and clinically useful results for physicians to make better decisions in their therapeutic measures.

Keywords: Hepatitis C;Genotype;Hemophilia;Iran
